# The Impact of Restrictive Policies on Mexican Immigrant Parents and Their Children's Access to Health Care

**DOI:** 10.1089/heq.2020.0111

**Published:** 2021-09-14

**Authors:** Abraham Aragones, Carolina Zamore, Eva M. Moya, Jacquelin I. Cordero, Francesca Gany, Denise M. Bruno

**Affiliations:** ^1^ Department of Psychiatry and Behavioral Sciences, Immigrant Health and Cancer Disparities, Memorial Sloan-Kettering Cancer Center, New York, New York, USA.; ^2^ Department of Social Work, College of Health Sciences, The University of Texas at El Paso, El Paso, Texas, USA.; ^3^ Department of Social Work, Border Biomedical Research Center, The University of Texas at El Paso, El Paso, Texas, USA.; ^4^ Community Health Sciences, SUNY Downstate School of Public Health, Brooklyn, New York, New York, USA.

**Keywords:** children, health care access, health disparities, immigrant parents, Latinos, primary care

## Abstract

**Background:** This study assessed whether policies that limit Mexican immigrants' access to care affects their children's access to a regular source of care, health insurance, and timely preventive health visits.

**Method:** This was a cross-sectional study among Mexican immigrant parents who attended a health promotion program in Texas, Nevada, New York, and Illinois. A sociodemographic survey, including parental and child variables, was administered.

**Results:** Children of parents without health insurance were almost four times more likely to be uninsured and eight times more likely to lack a regular source of care. Children of parents without a regular source of care were less than half as likely to have their own regular source of care than children whose parents had a regular source of care.

**Discussion:** Findings suggest when parents are uninsured/lack a regular source of care, a child's health disparity is created. Reducing disparities in health care coverage, affecting foreign-born parents, positively impacts their children's access to care.

Clinical Trial Registration number: NCT03209713.

## Introduction

Numerous studies have demonstrated the importance of accessible and high-quality primary care to the overall health and wellbeing of children.^1–5^ Children's insurance status is a strong predictor of their health care access as uninsured children have fewer visits to physicians, receive inadequate preventive services, and are more likely to be without a usual source of care.^6,7^ Continuous or regular contact with a provider or primary care facility (having a “medical home”) has been associated with positive health indicators, including increased use of preventive services, decreased use of emergency services, and shorter hospital stays.^8–10^ Regardless of whether the child(ren) of immigrants are born inside or outside the United States, they experience reduced odds of having a medical home, overall have less access to health care, and receive lower-quality care than children with U.S.-born parents.^8,11^

### Children's access to health care

A child's access to health care often results from a complex interplay of systemic factors and personal factors, while family immigration status serves as a key social determinant of health. The immigration status (i.e., unauthorized or type of authorization) of immigrant parents in the United States can determine their children's eligibility for and use of health care and consequently, their health outcomes.^11,12^ Therefore, national immigration policies impact the health of both foreign- and U.S.-born children of immigrant parents. Moreover, the impact of immigration status on child health can vary from state to state depending on the local health care access environment. Thus, it is critical to assess the effects of national immigration policies on children's access to health care and how these are mediated by state and local factors.

### National and state policy challenges to health care access

U.S. immigrants have lower rates of health insurance, usual source of care, and health care utilization than the general population, resulting in delayed treatment or failure to access care. Rates are significantly lower among recent immigrants and those without legal immigration status.^13–15^ Ample evidence indicates that state policies expanding health insurance eligibility to all groups, regardless of immigration status or date of entry to the United States, improve health care utilization and health outcomes among immigrants.^16–19^ Although the Patient Protection and Affordable Care Act (A.C.A.) increased health insurance access for immigrants, recent (<5 years in the United States) and undocumented immigrants cannot buy health insurance through the A.C.A. health exchanges. Additionally, under the Children's Health Insurance Program (CHIP) Reauthorization Act of 2009, most states currently allow lawfully residing immigrant children to receive Medicaid and/or coverage managed through state CHIP without the typical 5-year waiting period governing access to some other federal programs. However, variations in state-determined income eligibility scales increase disparities in access to such programs for immigrants throughout the United States. Currently, only the District of Columbia, California, Illinois, Massachusetts, New York, Oregon, and Washington offer state-only funded health coverage to children, regardless of immigration status.^20^

### U.S. immigrant population and health care status

Immigrants comprise a large and exponentially growing segment of the U.S. population, and changes to health policies can have broad ramifications to various immigrant groups. Immigrant numbers reached an all-time high of 44.5 million in 2017, accounting for 13.7% of the total U.S. population.^21^ Furthermore, 19.7 million people (34% of the total immigrant population) identify as Hispanic or Latino.^21^ In 2017, 18.2 million children in the United States (26% of the total U.S. population under 18) had immigrant parents.^21^ Of these, 10 million were citizen children with a noncitizen parent.^22^ Noncitizens, including lawfully present and undocumented immigrants, are more likely to be uninsured than citizens.^22^ Citizens with higher uninsured rates are often related to limited access to employer-sponsored health coverage and eligibility restrictions for Medicaid, State Children Health Insurance Program (SCHIP), and insurance options available on the A.C.A. marketplace. Whether eligible or otherwise, families with mixed legal status face barriers to enrollment, including language barriers, fear, confusion about policies, and difficulty navigating the enrollment process.^22^

### Objective

In this cross-sectional study of immigrant families, first we assessed whether parental access to care (having a regular source of care and/or health insurance) was associated with their child's (1) access to a regular source of care, (2) status of health insurance, and (3) timely preventive health visits among Latino/Hispanic children of Mexican immigrant parents in four states (New York, Texas, Illinois, and Nevada). These states differ in policies that determine eligibility for health/medical insurance and access to care for immigrants. Second, we assessed whether state policies regarding income eligibility requirements for Medicaid/CHIP and the availability of health insurance for immigrant children without legal status were associated with differences in access to care for this population. In addition, we also assessed whether a child's place of birth (inside/outside the U.S.) affected their health care access. Understanding access to care and health outcomes for mixed immigration status families can provide critical information to inform future policy, particularly in a political environment that threatens to reduce access to care for U.S. immigrants.

## Methods

We conducted a cross-sectional study among diverse self-identified Mexican immigrant parents of children ages 11 through 17 years who attended a health promotion program managed through their local Mexican consulate, in four geographically distinct cities in the United States: El Paso (TX), Las Vegas (NV), New York (NY), and Chicago (IL). The Institutional Review Boards at Memorial Sloan Kettering Cancer Center, the University of Texas at El Paso, and Biomedical Research Alliance of New York (B.R.A.N.Y.) approved this study and it is registered with clinicaltrials.gov.

Self-described Mexican-origin parents were approached to participate in the study managed by their local Mexican Consulate's Ventanilla de Salud (V.D.S.) (Health Window) program. The V.D.S. program operates in 50 Mexican consulates across the U.S. and is a collaboration between the Mexican government, nonprofits, and private agencies aimed at facilitating access to local health services while promoting a culture of health. They serve diverse Mexican populations, varying in age, years in the United States, Mexican regional origin, and reasons for and methods of migration. Moreover, these populations experience diverse health care safety nets due to regional/geographical location.

### Parent study

This is a nested cohort study within a more extensive randomized control trial evaluating the effectiveness of a community-based educational program to facilitate the completion of the human papillomavirus (HPV) vaccination series among children of Mexican or Mexican American parents (NIH/NIMHD-R01MD011508). Parents were recruited from November 2016 to May 2019. Parents who had at least one child ages 11 to 17 years, who had not previously received/completed the HPV vaccine series, were 21 years or older, self-identified as Mexican or Mexican American, and reported Spanish as their primary language were invited to participate in the study. The inclusion criteria consist of only one adult per family, who self-identified as a primary health caregiver. We specifically recruited the parent who self-described as the one managing all aspects of the child's health care needs. Parents who reported having multiple children were asked to provide information related to the youngest child eligible to receive the HPV vaccine at the time of recruitment.

### Recruitment procedures

During V.D.S. opening hours, bilingual Community Health Workers (CHWs) approached adults participating in V.D.S.-sponsored programs/services to assess eligibility. Once a participant was identified as eligible and willing to participate, verbal consent procedures followed. All materials and information were administered in Spanish. Participants were asked to complete a 5- to10-minute survey that included sociodemographic about themselves and their vaccine-eligible child. The questionnaire was administered using Research Electronic Data Capture (REDCap), a data management software system hosted and managed by Memorial Sloan Kettering.

### Variables

The primary outcomes evaluated children's health insurance status, presence/absence of regular sources of care, frequency of preventive provider visits, and date of most recent provider visits.

### Measurement

We obtained parental variables, including demographic characteristics, such as gender, age, level of education, duration of residence in the United States, and length of residence in their current state. Specifically, parents were asked if they had a personal doctor, clinic, or hospital where they receive regular health care; if they had health insurance and what type of insurance; as well as the same information for their study eligible child. Further questions about child's access to care were asked and included last time child visited regular source of care (for other than urgent/emergency purposes) and how often the child was taken to the regular source of care for a wellness visit (if a source of health care was identified). In addition, income eligibility criteria for Medicaid/SCHIP and the availability of health insurance for children without legal immigration status in the four states were assessed.^20^ We analyzed the data using S.P.S.S. Statistics 26. Descriptive statistics were used to evaluate demographic variables such as education, length of stay in the United States, length of stay in the state, and medical insurance type. Associations between parent and child access to care were explored, and their respective odds ratios (ORs) were reported.

## Results

### Descriptive statistics

Among 415 self-described Mexican immigrant parents in the study, 123 (30%) lived in El Paso, TX; 121 (29%) lived in Chicago, IL; 96 (23%) lived in New York, NY; and 75 (18%) lived in Las Vegas, NV. All parents were born in Mexico, and most (89%) reported having lived in the United States for more than 5 years. The average age of parents in the study was 40.3 years, and all were self-reported as the child's main caregiver; 391 (94%) were female. Other characteristics, by city, are described in Table 1.

**Table 1.** Characteristics of Parents in the Study

	Chicago, %	El Paso, %	Las Vegas, %	New York City, %
Parent sex				
Female	91	96	92	94
Education				
<8th grade	40	49	46	58
≥8th grade	60	51	54	42
Language preferred				
Spanish	100	100	100	100
Years since migrated to the U.S.				
<5 years	2	19	19	3
6–9 years	3	11	8	3
10–15 years	27	21	25	29
>15 years	68	49	48	65
Years living in current state				
<5 years	6	21	20	4
6–9 years	4	11	8	6
10–15 years	20	24	24	29
>15 years	70	44	48	61

Overall, 25% (103) of the reporting parents in the study reported having health insurance, of those, 76% reported having a public health insurance plan (Medicaid or state plan). Fifty-seven percent (235) of parents reported having a regular source of care, almost half (46%) of whom reported having health insurance coverage. There was a high level of having a regular source of care among parents with health insurance at 90%. Significant differences in rates of parental insurance coverage were observed between cities, with New York City having the highest rate at 35%, followed by El Paso at 29%, Chicago at 22%, and Las Vegas at 9% (*p*=0.001). Significant differences were also observed in the proportion of parents reporting a regular source of care: 76% in New York City, 71% in Chicago, 51% in El Paso, and 17% in Las Vegas (*p*<0.001).

Seventy-eight percent of parents reported having health insurance coverage for their child, of whom 90% were covered by a public health insurance plan, such as SCHIP/Medicaid). Most parents (85%) reported having a regular source of health care for their children. Most (329 [79%]) of the children assessed in the study were born in the United States. Eighty-six percent of the U.S.-born children were reported to have health insurance, which was almost double the insurance rate of the Mexico-born children at 45% (*p*<0.001). Furthermore, having a regular source of care was more frequent among children born in the United States (90%) than among children born in Mexico (63%) (*p*<0.001). Differences observed among the four cities showed 95% of parents in New York City and 89% in Chicago reported having health insurance for their child versus 63% in El Paso and 60% in Las Vegas (*p*<0.001). Similar differences were found regarding children's regular source of care, as 96% of parents in New York City and 95% in Chicago reported having a regular source of care for their children versus 80% in El Paso and 61% in Las Vegas (*p*<0.001).

Seventy-seven percent of parents reported that their children's most recent health care provider visit (other than for an emergency) occurred during the previous 12 months. Still, there were significant differences between sites: 84% in New York City, 83% in Chicago, 74% in El Paso, and 63% in Las Vegas (*p*=0.002). Overall, 73% of parents reported taking their child for an annual wellness visit, but there was a significant difference in the rate between those who did or did not have a regular source of care: 93% versus just 7%, respectively (*p*<0.001). Of the parents who reported an annual wellness visit, significant differences were found between cities: 88% in New York City, 79% in Chicago, 64% in El Paso, and 59% in Las Vegas, *p*<0.001.

Children of parents without health insurance were almost four times more likely to be uninsured (OR, 3.85; 95% confidence interval [CI], 1.86–7.97) and over eight times more likely to lack a regular source of care (OR, 8.1; 95% CI, 2.48–26.42). Uninsured children in the study were significantly less likely to have had an annual wellness visit (OR, 0.1; 95% CI, 0.12–0.32) and less likely to have visited a health care provider in the previous 12 months (OR, 0.16; 95% CI, 0.1–0.27). Similarly, children of parents without a regular source of care were less than half as likely to have their own regular source of care than children whose parents had a regular source of care (OR, 0.4; 95% CI, 0.21–0.78). A child lacking a regular source of care reduced their odds of having an annual health care provider visit (OR, 0.11; 95% CI, 0.06–0.21) and having visited a health care provider during the previous 12 months (OR, 0.08; 95% CI, 0.04–0.15).

Children of participants residing in states with health insurance available for children in mixed legal status households and with higher CHIP/Medicaid income eligibility thresholds (NY and IL) were almost seven times more likely to have health insurance than those living in states without (TX and NV) (OR, 6.74; 95% CI, 3.85–11.82). Other factors associated with increased odds of a child having health insurance included the child's country of birth, with U.S.-born children being more than seven times as likely as foreign-born children to have it (OR, 7.41; 95% CI, 4.37–12.55). Parents who lived in their current state of residence for over 5 years were almost four times as likely to have health insurance for their children than those who had lived in their state for less than 5 years (OR, 3.90; 95% CI, 2.09–7.28). Factors associated with increased odds of a child having a regular source of health care included parents having lived in the United States (OR, 4.52; 95% CI, 2.28–8.95) or their current state of residence (OR, 3.73; 95% CI, 1.91–7.26) for more than 5 years.

## Discussion

Increased access to quality health care among all children, including Latino children, is strongly associated with positive health outcomes and with reducing or eliminating health disparities.^8–10^ In our current study assessing baseline data, we found children's access to care was highly associated with their respective immigrant parents' access to care. Specifically, children whose parents reported being uninsured or lacking a regular source of care were more likely to lack insurance, a regular source of care, a provider visit in the previous 12 months, or annual wellness visits. Having a regular source of care was significantly associated with having annual wellness visits, and uninsured children were significantly less likely to have annual wellness visits. Furthermore, foreign birth was significantly associated with lack of health insurance and a regular source of care, and U.S.-born children of immigrants were far more likely to have health insurance than their foreign-born peers.

Over 18 million children in the United States live in households with at least one immigrant parent, accounting for over a fourth (26%) of the total child population in the United States.^23^ In addition, the fastest-growing child population by race/ethnicity is Latino, almost doubling in size during the previous decade, with the most being of Mexican origin or ancestry.^20^ Previous findings on Latino parents suggest higher rates of being uninsured in the United States, consequently affecting their and their U.S.-born children's health care access, utilization, and health outcomes.^6^ Assessing the implications of access-to-care disparities observed among Latino immigrant parents, particularly in a political environment with the potential to create further access barriers, is imperative for these children's health. Our current study suggests that uninsured Latino children, as well as Latino children without a regular source of care, were less likely to visit a health care provider, reducing the chances for timely preventative care, such as immunizations, periodic screenings, diagnosis, and treatment. In our study, Latino children of immigrant parents were less likely to have health insurance, less likely to have a regular source of care, and less likely to report visiting their doctor for preventive visits annually if the parent reported a lack of health insurance and a regular source of care. Policies that further restrict access to care like expansion of public charge criteria, immigration status, and family income will worsen health disparities in marginalized and minority populations. Federal, state, and/or local policies could instead focus on reducing disparities that affect children's health since, for many conditions, childhood health predicts health in adulthood.^24^ Poor health (lacking a medical home, undiagnosed/untreated health conditions) in childhood can also limit educational attainment, which also adversely affects adult health.^24^

The current study found significant differences in parental insurance and regular source of care rates among the four study sites. Parents from El Paso and Las Vegas had lower rates of both insurance and regular source of care than parents from New York City and Chicago. The difference in rates between states is correlated to significant differences in eligibility for Medicaid/CHIP, the most common type of child health insurance reported by parents in our study. For example, NY and IL have a significantly higher income eligibility threshold than TX and NV. New York and IL are among the few states to use state-only funds to cover eligible children, regardless of immigration status.^24^ Based on these differences, we compared access-to-care rates for parents and children grouped by higher income eligibility thresholds for Medicaid/CHIP and availability of health insurance for children regardless of legal immigration status versus those with lower income eligibility thresholds and availability of health insurance according to legal immigration status (e.g., NY and IL vs. TX and NV). Findings indicated that children in the study who lived in NY and IL were significantly more likely to have health insurance and to have a regular source of care than those who lived in TX and NV.

Study findings reveal disparities resulting from differences in insurance eligibility policies for adults, children, and families. Our study supports the growing evidence that policies expanding eligibility or availability of insurance for children and families have a significant effect on children's access to care.^9^ Furthermore, evidence shows that punitive immigration policies impact children's health.^24^ Several policies related to immigration and health are currently under review, such as limiting family reunification and immigration and broadening public charge criteria. It is imperative that we understand and are mindful of the potential negative upstream consequences of these policies on the health and wellbeing of vulnerable populations. As our study demonstrates, policies affecting the health insurance eligibility or the access to care of immigrant parents are related to children's health but further research is needed to disentangle how a state's polices directly interact with parental health insurance and hence children's access to care.

There are some limitations to our study. The study design relies on self-reported information for access to and use of health care and insurance status and may be affected by recall bias. Also, the sample included predominantly female caretakers. Although “mothers” in this population are critical in household production of health, obtaining information from one parent might limit our findings' generalizability. Future research should assess both parents' access to health care for a more granulated understanding of its associations with a child's access to care. Additionally, children who had previously received/completed the HPV vaccine were excluded from this study, which may have affected our results. Despite these limitations, this study gives a critical look at the barriers imposed by state policies on immigrant parents' health care access and how it affects their children's access to health care.

The interplay of immigration (e.g., country of origin, legal status), parental access to care, and children's access to care are complex. Other factors, including time in the United States, language and English proficiency, ethnicity, and household income, in addition to immigrant status, are key in access to and utilization of quality health care.^13,15^ Despite these multiple factors, we believe focusing in a subset of such factors is imperative to creating positive upstream access-to-care outcomes. These factors include health insurance and access to care for all children residing in the United States. In conclusion, understanding the associations between parental and child access to care is critical to improve health equity and inform policies, reduce health disparities, and improve health outcomes in childhood and, subsequently, adulthood.

## Abbreviations Used

A.C.A.: Affordable Care Act
CHIP: Children's Health Insurance Program
CI: confidence interval
HPV: human papillomavirus
OR: odds ratio
REDCap: Research Electronic Data Capture
V.D.S.: Ventanilla de Salud
